# Post-COVID cognitive dysfunction in socioeconomically-vulnerable older adults: findings from a Brazilian cohort

**DOI:** 10.1055/s-0046-1816035

**Published:** 2026-02-27

**Authors:** Danielle Calil de Sousa, Arnaldo Santos Leite, Luiz Gustavo Guimarães Sacramento, Bruna Achtschin Fernandes, Bárbara Caroline Dias Faria, Carolina Coimbra Marinho, Paulo Caramelli

**Affiliations:** 1 Universidade Federal de Minas Gerais, Faculdade de Medicina, Belo Horizonte MG, Brazil.; 2 Faculdade Ciências Médicas de Minas Gerais, Faculdade de Medicina, Belo Horizonte MG, Brazil.

**Keywords:** COVID-19, Post-Acute COVID-19 Syndrome, Cognitive Dysfunction

## Abstract

**Background:**

Cognitive impairment is increasingly recognized as a long-term consequence of coronavirus disease 2019 (COVID-19), but most evidence comes from high-income settings. Little is known about its impact in socioeconomically-vulnerable populations.

**Objective:**

The current study investigated long-term cognitive effects in 133 individuals older than 50 years of age with low level of schooling and low socioeconomic status who were hospitalized for COVID-19.

**Methods:**

The participants were assessed 12 to 18 months after hospitalization using the Modified Telephone Interview for Cognitive Status (TICS-M). A subset of 65 participants underwent further cognitive and neuropsychiatric evaluations. Cognitive impairment was defined as scores ≤ −1.5 standard deviations from age- and education-adjusted Brazilian norms. During the acute phase of the disease, sociodemographic, clinical, and laboratory data were evaluated to identify potential risk factors.

**Results:**

The mean age of the subjects (n = 65) was of 65.3 years, and the sample was composed of 69.2% of women, 57.1% of pardo individuals, 47.7% of subjects with ≤ 4 years of schooling, and 83.6% of participants with monthly family income ≤ 3 minimum wages. Hospitalization averaged 16.1 days, and 55.4% required intensive care. Cognitive impairment affected 70.8% of the participants. Higher age, female sex, and hyposmia were associated with cognitive impairment.

**Conclusion:**

Cognitive impairment was frequent in this socioeconomically-vulnerable sample, a group still underrepresented in existing research.

## INTRODUCTION

Coronavirus disease 2019 (COVID-19) shows persistence of many symptoms after the acute phase of the infection. A wide range of persistent symptoms, including cognitive complaints, have been reported after acute COVID-19. 1 Due to the heterogeneity of these symptoms and the difficulty in attributing them to the infectious context itself, the post-COVID-19 state is not fully understood, including its cognitive manifestations.

Long-term cognitive dysfunction has increasingly been recognized as a core component of the post-COVID-19 condition. Previous studies 2 have indicated that individuals may experience cognitive deficits for months after the acute infection. The most frequently affected domains include attention, memory, and executive functions, although difficulties in language and visuospatial abilities have also been described. However, estimates remain inconsistent due to heterogeneity in diagnostic definitions, neuropsychological instruments, and follow-up intervals across studies. Moreover, sociodemographic variability, differences in health care systems, and policies related to quarantine and vaccination further complicate cross-study comparisons. 2

Recent evidence shows that cognitive impairment has been frequently observed and persistent after COVID-19 infection; A recent mixed-methods study in the United Kingdom 3 found that individuals with post-COVID-19 condition frequently report memory, attention, language, executive, and processing-speed difficulties, with symptoms lasting up to 2 years and significantly affecting daily functioning and mental health.

Most of the studies investigating cognitive impairment after COVID-19 published so far have included people with medium or high levels of schooling, as well as middle or high socioeconomic status. Hence, investigation of long-term cognitive complaints in the post-COVID-19 population becomes relevant, especially in populations that have been poorly represented in published studies. The aim of the present study was to investigate long-term cognitive and neuropsychiatric features in individuals with low level of schooling and low socioeconomic status who were hospitalized for COVID-19.

## METHODS

### Setting

We conducted a cross-sectional analysis of a cohort of individuals hospitalized with moderate or severe forms of COVID-19 in the city of Belo Horizonte, state of Minas Gerais, Brazil. The cohort consisted of participants aged ≥ 18 years with hospitalization for COVID-19 confirmed by reverse-transcription polymerase chain reaction (RT-PCR) positivity for severe acute respiratory syndrome coronavirus 2 (SARS-CoV-2) in nasal/oropharyngeal swabs or tracheal aspirates with severity criteria for SARS. These participants had a history of hospitalization at three public hospitals affiliated with the Brazilian Unified Health System (Sistema Único de Saúde, SUS, in Portuguese): Hospital das Clínicas da Universidade Federal de Minas Gerais, Hospital Eduardo de Menezes, and Hospital Júlia Kubitschek.

### Participants

The participants were recruited between June 2020 and April 2021 at the 3 participating hospitals. Sociodemographic, clinical, and laboratory data were collected during the participants' hospitalization in the acute phase of COVID-19.

One year after hospitalization for COVID-19, through outpatient follow-up, individuals aged ≥ 50 years were invited to participate. The age range was chosen based on the available Brazilian normative data for the cognitive battery used in the study, as well as the observation of greater long-term deleterious effects on cognition in older individuals. 4 Subjects with a previous diagnosis of dementia of any etiology or previous stroke were excluded from the evaluation based on a clinical interview.

The study was approved by the Research Ethics Committee of Universidade Federal de Minas Gerais (under CAAE: 30415820.6.0000.5149), and it was conducted according to the ethical standards of the Declaration of Helsinki. All participants signed the written informed consent form.

### Cognitive and neuropsychiatric assessments

Individuals under outpatient follow-up took part in a telephone cognitive screening using the Modified Telephone Interview for Cognitive Status (TICS-M). After the initial assessment, they were invited to in-person cognitive and neuropsychiatric assessments. The participants were called in alternation: assessment of participants with altered TICS-M and with normal TICS-M. The protocol for the cognitive and neuropsychiatric assessments lasted approximately 60 minutes, and it consisted of the following tests and scales: the Addenbrooke's Cognitive Examination-Revised (ACE-R), 5 6 the Picture-Free and Cued Selective Reminding Test (P-FCSRT), 7 the Hospital Anxiety and Depression Scale (HADS), 8 and the Impact of Events Scale-Revised (IES-R), 9 which were administered to each participant, and the Functional Activities Questionnaire (FAQ), 10 which was administered to a an individual with close ties to the participant. The analysis of the cognitive scores was based on age- and education-adjusted normative data available for the Brazilian population. 5 6 7 The cognitive and neuropsychiatric assessments were conducted by a single researcher (DCS) to ensure standardization and minimize inter-rater variability.

### Statistical analyses

Cognitive impairment was defined as a performance of ≤ −1.5 standard deviations in any cognitive domain (attention, executive function, memory, visuospatial ability, and language) on the ACE-R or in the delayed free and total recall on the P-FCSRT. The ACE-R cut-off was based on age- and education-adjusted normative data available for the Brazilian population, and the delayed free and total recall P-FCSRT cut-off used was also from a Brazilian study. 5 6 7 The use of a cutoff of −1.5 standard deviations below normative values in at least 1 cognitive domain is widely accepted in clinical and research settings for the definition of cognitive impairment, such as in the diagnostic framework for mild cognitive impairment. 11 Neuropsychiatric symptoms such as anxiety, depression and posttraumatic stress disorder were assessed using the cut-off points suggested by the HADS and IES-R scales.

Sociodemographic, clinical, and laboratory data during the acute phase of SARS-CoV-2 infection were analyzed as potential risk factors for cognitive impairment after 1 year of COVID-19. A logistic regression model was used for this evaluation. Univariate analysis was conducted using Mann-Whitney U tests, independent samples *t* -tests, Chi-squared (χ ^2^ ) tests, and Fisher's exact tests. Variables with a significance level of up to 0.25 in the univariate analysis were selected for inclusion. The statistical analyses were performed using the IBM SPSS Statistics for Windows (IBM Corp.) software, version 23.0.

## RESULTS

During the recruitment period, 427 individuals were hospitalized with COVID-19. A total of 106 were excluded because they were under 50 years of age, and 5, due to preexisting diagnoses of dementia or stroke. Therefore, 321 participants were invited to enroll in the study.

Throughout the 1-year follow-up, 25 participants died, 4 withdrew consent, and 133 were lost to follow-up. Thus, 160 participants aged ≥ 50 years were invited to undergo the TICS-M telephone cognitive screening battery. In this sample, 3 participants withdrew consent and 24 were lost to follow-up. Of the 133 individuals who underwent the screening test, 19 (14.3%) had altered TICS-M and 114 (85.7%), normal TICS-M.

**Figure 1:**
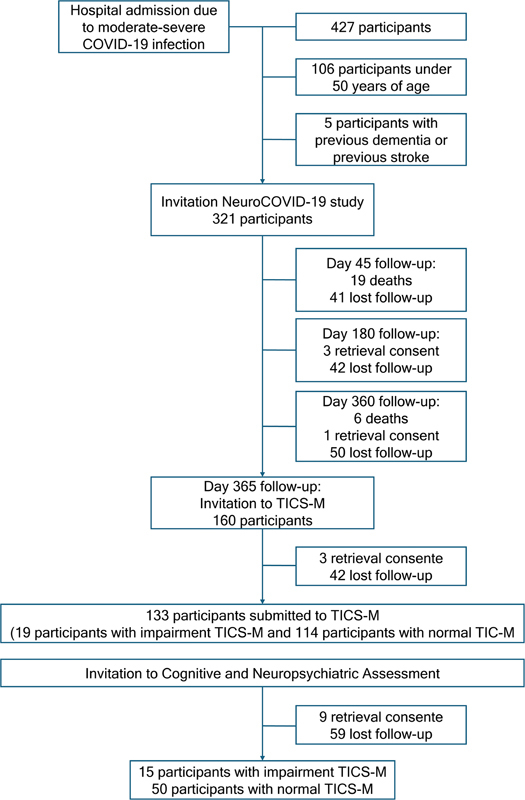
Note: Between June 4, 2020, and April 20, 2021. Flowchart of the sampling design of the study.

Between 12 and 18 months after the hospitalization, the subjects were invited to participate in the cognitive and neuropsychiatric assessments. During this period, 65 participants were assessed: 15 with altered TICS-M and 50 with normal TICS-M. The flowchart in Figure 1 shows the study's sampling design.

### Baseline characteristics

The sample (n = 65) had a mean age of 65.3 ± 8.0 years, 69.2% were women, 57.1% self-identified as pardo, 47.7% had less than 5 years of schooling, and 83.6% had a monthly family income lower than USD 687, which corresponds to 3 minimum wages in Brazil.

The mean length of hospital stay was of 16.1 ± 10.4 days; 55.4% of the participants required admission to intensive care unit (ICU), with an average length of stay of 9.5 ± 7.3 days. Mechanical ventilation was required in 27.7% of the patients. Acute respiratory distress syndrome (ARDS) was diagnosed in 4.7% of the patients, with use of vasopressors in 14.5% and use of inotropes in 4.8% of the cases.

In the acute phase of COVID-19 infection, hyposmia was reported in 34.4% of the patients, dysgeusia, in 39.1%, and headache, in 20.3%. Hypertension was the most common comorbidity (75.4%), followed by obesity (41.5%) and diabetes mellitus (38.5%).

Tables 1 and 2 depict the sociodemographic and clinical-laboratory profiles of the study participants.

**Table 1:** Sociodemographic characteristics of the study sample

Sociodemographic characteristics (n = 65)		Results
**Age (years)**	Range	50.0–85.0
	Mean ± standard deviation	65.3 ± 8.0
	Median (Q25–Q75)	64.0 (60.0–71.0)
**Sex: n (%)**	Female	45 (69.2%)
	Male	20 (30.8%)
	Total	65 (100.0%)
**Self-declared race/skin color: n (%)**	Black	13 (20.6%)
	Pardo	36 (57.1%)
	Indigenous	2 (3.2%)
	White	11 (17.5%)
	Yellow	1 (1.6%)
**Family income (BRL): n (%)**	None	1 (1.8%)
	Below 1 minimum wage	1 (1.8%)
	Between 1 and 3 minimum wages	44 (80.0%)
	Between 4 and 5 minimum wages	6 (10.9%)
	Between 6 and 15 minimum wages	3 (5.5%)
**Schooling (years): n (%)**	0	4 (6.2%)
	1–4	27 (41.5%)
	5–8	14 (21.5%)
	9–11	13 (20.0%)
	≥ 12	7 (10.8%)

Abbreviations: BRL, Brazilian real; Q25, 25th quartile; Q75, 75th quartile.

**Table 2:** Characterization of patients according to clinical data

Clinical information (n = 65)			Results
**Length of hospital stay**	Range	3.0–53.0
	Mean ± standard deviation	16.1 ± 10.4
**ICU: n (%)**		36 (55.4%)
**Length of ICU stay**	Range	1.0–32.0
	Mean ± standard deviation	9.5 ± 7.3
**Mechanical ventilation: n (%)**		18 (27.7%)
**ARDS: n (%)**		3 (4.7%)
**Renal replacement therapy: n (%)**		1 (1.54%)
**Vasopressors: n (%)**		9 (14.5%)
**Inotropics: n (%)**		3 (4.8%)
**Comorbidities: n (%)**	Hypertension	49 (75.4%)
	Diabetes mellitus	25 (38.5%)
	COPD	2 (3.1%)
	Obesity	27 (41.5%)
	Chronic kidney disease	4 (6.3%)
	Others	22 (33.8%)
**D-dimer**	Range	173.0–69.981.0
	Mean ± standard deviation	5787.2 ± 14.356.1
**CRP**	Range	0.0–343.0
	Mean ± standard deviation	100.8 ± 76.1
**Lymphocytes**	Range	0.0–118.0
	Mean ± standard deviation	5.9 ± 21.0
**PaO2/FiO2 ratio: n (%)**	< 100 with ventilatory support	2 (3.4%)
	< 200 with ventilatory support	10 (16.7%)
	< 300	26 (43.3%)
	300–399	11 (18.3%)
	> 400	11 (18.3%)
**Hyposmia: n (%)**		22 (34.4%)
**Dysgeusia: n (%)**		25 (39.1%)
**Headache: n (%)**		13 (20.3%)

Abbreviations: ARDS, severe respiratory distress syndrome; COPD, chronic obstructive pulmonary disease; CRP, C-reactive protein; ICU, Intensive Care Unit; PaO2/FiO2 ratio, ratio of partial pressure of oxygen to inspired oxygen fraction.

A comparison was made in terms of sociodemographic and clinical characteristics to assess whether the sample of 65 participants was representative of the patients eligible for the study at the time of recruitment. The sample of 65 participants was found to have more women, to be more ethnically-diverse and less likely to have chronic obstructive pulmonary disease. Additional information is available in the **Supplementary Appendix** ( **Supplementary Material** – available at https://www.arquivosdeneuropsiquiatria.org/wp-content/uploads/2025/11/ANP-2025.0194-Supplementary-Material.docx ; **Tables S1–S4** ).

### Cognitive and neuropsychiatric assessments

The cognitive and neuropsychiatric assessments were performed at a mean of 14.6 (range 12–18) months after hospitalization. Overall, 25 participants of the sample (35.8%) had impaired global ACE-R. Cognitive impairment was detected in 70.8% of the sample.

The affected cognitive domains in these participants, in descending order of prevalence, were memory (53.8%), language (33.8%), executive function (27.7%), attention (26.2%), and visuospatial abilities (13.8%). These findings can be better evaluated in Table 3 .

**Table 3:** Characterization of the participants according to the results of the cognitive, neuropsychiatric, and functional tests

Cognitive and neuropsychiatric assessments (n = 65)		Results: n (%)
**Mini-Mental State Examination**	Impaired	7 (10.8%)
**ACE-R Global score**	Impaired	25 (38.5%)
**Cognitive domains**		
*Attention*	Impaired	17 (26.2%)
*Memory*	Participants with at least one memory task impaired	35 (53.8%)
	Only impaired in the ACE-R	5 (7.6%)
	Only impaired in the Delayed P-FCSRT-IR TSFT	7 (10.7%)
	Only impaired in the Delayed P-FCSRT-IR TST	4 (6.1%%)
*Executive Function*	Impaired	18 (27.7%)
*Language*	Impaired	22 (33.8%)
*Visuospatial Abilities*	Impaired	9 (13.8%)
**HADS: anxiety**	Impaired	24 (36.9%)
**HADS: depression**	Impaired	9 (13.8%)
**IES-R**	Impaired	23 (35.4%)
**FAQ**	Functionally disabled	2 (3.2%)
	Total	63 (100%)

Abbreviations: ACE-R, Addenbrooke's Cognitive Examination-Revised; FAQ, Functional Activities Questionnaire; HADS, Hospital Anxiety and Depression Scale; IES-R, Impact of Events Scale-Revised; P-FCSRT-IR, Picture Free and Cued Selective Reminding Test–Immediate Recall; Delayed P-FCSRT-IR TSFT, Picture Free and Cued Selective Reminding Test Immediate Recall–Total Sum of Free Trials; Delayed P-FCSRT-IR TST, Picture Free and Cued Selective Reminding Test Immediate Recall–Total Sum of Trials.

In the assessment of neuropsychiatric symptoms, 36.9% of the participants had symptoms of anxiety, 13.8%, of depression and 35.4%, subjective symptoms of posttraumatic stress disorder.

The functional assessment showed that 3.2% of the sample (n = 2) had significant impairment in activities of daily living based on the score on the FAQ scale, suggesting a diagnosis of mild dementia. This finding was not observed before the SAR-CoV-2 infection, according to the respondents, who had close ties with the patients. Among these two participants, one presented with elevated HADS-A and IES-R scores during the assessment, which may partly explain the observed findings. The other was the oldest participant in the cohort (aged 85 years). Although individuals with a prior diagnosis of dementia were excluded, and family members reported no previous functional decline, there were no formal medical records documenting prior cognitive status. It remains possible that this participant had already manifested clinical features consistent with mild cognitive impairment, a condition that is often underdiagnosed in low-income populations.

### Predictors of cognitive impairment after COVID-19

Variables from the acute phase of COVID-19 with a *p* -value ≤ 0.25 (age, sex, education, ICU admission, length of hospital stay, hyposmia, dysgeusia) on the univariate analysis were included in the multivariate analysis to assess their predictive value for cognitive impairment 12 to 18 months after COVID-19. An initial logistic regression model was developed using this selection of variables. The use of inotropic agents, although presenting a probability of significance lower than 0.25, was not included in this analysis due to the small number of patients who used this drug. In the final model, the variables that jointly influenced cognitive impairment were age, sex, hyposmia ( Table 4 ).

**Table 4:** Logistic regression model of factors associated with cognitive impairment (n = 65)

Variables		Final model		
		*β*	Odds ratio (95%CI)	*p*
**Age**		0.1	1.2 (1.0–1.3)	**0.007**
**Sex (reference: male)**	Female	1.7	5.7 (1.2–25.6)	**0.025**
**Hyposmia (reference: no)**	Yes	1.6	5.0 (1.1–23.7)	**0.040**

Note: Values of *p* in bold indicate statistical significance.

With increasing age, a patient's risk of developing cognitive impairment increases 1.2-fold. In the current study, women were 5.7 times more likely to present cognitive impairment than men, and the presence of hyposmia in the acute phase of COVID-19 infection in patients with moderate forms of infection was 5 times more likely to result in cognitive impairment than in a patient without hyposmia.

## DISCUSSION

Cognitive impairment after 12 to 18 months of the acute phase of COVID-19 in individuals aged ≥ 50 years was a frequent finding in the current study. The prevalence rates were also higher compared to those other studies 12 13 14 15 16 that assessed cognitive performance 1 year after the acute phase of COVID-19 infection. Assessment of the prevalence of cognitive impairment in individuals after COVID-19 infection is challenging because of the heterogeneity among studies in the definition of cognitive impairment, the timing of cognitive assessment after the acute phase of SARS-CoV-2 infection, and the heterogeneity in sociodemographic and clinical data of the samples studied. 2

Damiano et al. 17 also investigated cognitive and neuropsychiatric outcomes after COVID-19 in a large Brazilian cohort. Although that study did not estimate an overall prevalence of cognitive impairment, it reported a high frequency of subjective cognitive complaints (51%) and demonstrated deficits in cognitive test performance, particularly in the attention and executive function domains. These findings provide an important point of comparison with our results, although key methodological and demographic differences must be considered. In particular, the cohort assessed by Damiano et al. 17 was larger (n = 425), younger on average (mean age of 56 years), and evaluated 6 to 9 months after hospitalization, whereas our participants were older (mean age of 66 years) and underwent assessment 12 to 18 months after discharge. Both cohorts were characterized by low levels of schooling; in Damiano et al., 17 more than half of the participants had not completed elementary school, while our sample was further distinguished by low income and socioeconomic vulnerability, factors that may compound the risk of long-term cognitive impairment.

The high socioeconomic vulnerability of the sample, in which 47.7% had fewer than 5 years of schooling and 83.6% had a family income of up to 3 minimum wages, may have contributed to the high prevalence found in the current study. Previous studies 18 19 have shown that formal education plays a significant role in cognitive reserve, which is the ability to maintain cognition despite neuropathologic insults. Furthermore, previous studies 20 have also found that persistence of post-COVID-19 symptoms is associated with poorer socioeconomic conditions.

Another potential reason for the high prevalence is that the sample included only moderate and severe cases of COVID-19 who were hospitalized, with 55.4% of the participants requiring intensive care, 27.7% requiring mechanical ventilation, and 4.7% meeting criteria for ARDS. Severe forms of COVID-19 are associated with poorer cognitive performance than mild forms of the infection, according to previous studies. 21 22 23

Memory (53,8%), language (33.8%) and executive function (27.7%) were the most affected domains in the sample of the present study. Compared to previous investigations in which the most common impairment profile was attention, memory, and executive function, a different finding of the present study is the high rate of language impairment. The low level of schooling of the sample may be a reason for this finding when compared with the educational profile in the aforementioned studies, in which a large proportion of the samples had 12 or more years of schooling. Although the assessment of cognitive domains in the current study was based on normative values for age and education derived from tests conducted in Brazilian populations with diverse levels of schooling, language performance varies significantly across the regions of Brazil, influenced by factors such as the quality of the education, literacy habits, and professional occupations. 5 6 24 These heterogeneous influences may partly explain the high prevalence of language impairment observed in our sample.

Higher age, female sex and hyposmia in the acute phase of the SARS-CoV-2 infection were predictive factors of cognitive impairment 12 to 18 months later, findings similar to those of other studies. 4 17 25 Previous epidemiological data 11 suggests that the prevalence of cognitive impairment increases with age. Women have also been found to be associated with persistent executive dysfunction in previous studies. 4 Moreover, the association between olfactory dysfunction and cognitive changes has been found 26 in other neurological conditions (such as neurodegenerative diseases, multiple sclerosis, and traumatic brain injury). The association between hyposmia and cognitive impairment in the post-COVID-19 population has already been reported. 25 In a previous systematic review, 26 the association between olfactory dysfunction and cognitive impairment after COVID-19 was suggested to be more related to intrinsic associations between olfaction and cognition in humans than to the mechanism of direct virus entry into the central nervous system.

Anxiety (36.9%), depression (13.8%) and posttraumatic stress disorder (35.4%) were also common in the sample of the present study. This prevalence was also higher than in previous studies. 17 The socioeconomic vulnerability of the study participants may also explain this finding. There is previous evidence 27 of associations involving mental disorders and socioeconomic gradients such as low income, financial hardship, low schooling, professional occupation, and unemployment.

The aim of the current research was to study the long-term cognitive effects of post-COVID-19 condition and its potential risk factors in a sample that is vulnerable from socioeconomic and clinical points of view. In addition, the assessment of cognitive impairment included a comprehensive evaluation of different domains (such as attention, memory, executive function, language, and visuospatial ability) and considered impairment in a more accurate way, by using cut-off points related to normative data of a specific population.

We acknowledge that the present study has several limitations. Although the sample size was small, some studies in this field 12 28 29 have reported similar sizes. The sample was collected on a convenience basis, which limits the statistical robustness of the results at the population level. The possibility of selection bias cannot be ruled out, as the participants most likely to be interested in participating in the research were those with a possible preexisting cognitive complaint. Nonetheless, we attempted to reduce this bias by alternating the invitation of individuals with both normal and altered TICS-M scores for the in-person assessment. In addition, our comparison between the final evaluated sample and the broader eligible population ( **Supplementary Material Tables S1–S4** ) showed a largely comparable profile, supporting the representativeness of the sample within the study's scope. Another limitation to consider is that memory may appear more impaired because it was assessed with two instruments (ACE-R and P-FCSRT). However, this methodological choice reflected our intention to explore memory in greater detail, as it is among the most frequently affected cognitive domains in individuals with long COVID. Moreover, the P-FCSRT can be administered with relative ease even to cognitively-healthy individuals with very low schooling, which makes it particularly suitable for our sample.

One of the study's exclusion criteria was a previous diagnosis of dementia according to the report by an individual with close ties to the participant, although there was no formal documentation of these participants' previous cognitive status. However, this is also a limitation of other studies in this field. 13 22 30 A control group for the study was not available at that time, which is another limitation that was present in previous studies. Because not all participants had an individual with close ties who lived with them prior to their admission due to COVID-19, it was not possible to use a retrospective dementia diagnosis tool, such as the Informant Questionnaire on Cognitive Decline in the Elderly (IQCODE). Moreover, the current analysis did not include complementary testing, such as neuroimaging, and genetic or biomarker evaluations to better define the etiologies of cognitive impairment. While the lack of retrospective cognitive assessments is a limitation, this approach aimed to prioritize feasibility and inclusivity in a population with reduced healthcare access.

The use of the TICS-M enabled the initial cognitive screening in a standardized and scalable manner, appropriate for pandemic-era constraints and populations with reduced digital literacy. The subsequent in-person assessments followed a structured protocol that minimized assessment bias by including TICS-M-normal and TICS-M-abnormal participants in alternation.

Despite these limitations, the current study contributes valuable data from an underrepresented population that faces structural barriers to healthcare and research participation. Most post-COVID cognitive studies focus on individuals with higher education and socioeconomic status, which may mask the true burden of long-term cognitive impairment in vulnerable groups. Our findings underscore the need for inclusive research strategies that reflect the broader impact of COVID-19.

The present study shows a significant rate of cognitive impairment 12 to 18 months after the acute phase of moderate and severe forms of COVID-19 infection. Higher age, female sex, and hyposmia were clinical factors associated with the outcome of cognitive impairment. Future studies with larger sample sizes and clinical assessment using biomarkers are essential for a better interpretation of these data.
